# Pre-Clinical and Clinical Advances in Gene Therapy of X-Linked Retinitis Pigmentosa: Hope on the Horizon

**DOI:** 10.3390/jcm14030898

**Published:** 2025-01-29

**Authors:** Nadezhda A. Pechnikova, Malamati Poimenidou, Ioannis Iliadis, Maria Zafeiriou-Chatziefraimidou, Aleksandra V. Iaremenko, Tamara V. Yaremenko, Kalliopi Domvri, Alexey V. Yaremenko

**Affiliations:** 1 Department of Biochemistry and Biotechnology, University of Thessaly, 38446 Volos, Greece; npechnikova@uth.gr; 2 Laboratory of Chemical Engineering A’, Department of Chemical Engineering, Faculty of Engineering, Aristotle University of Thessaloniki, 54636 Thessaloniki, Greece; 3 Saint Petersburg Pasteur Institute, Saint Petersburg 197101, Russia; 4 School of Medicine, Aristotle University of Thessaloniki, 54124 Thessaloniki, Greece; matina.poim@gmail.com (M.P.); johniliadis96@gmail.com (I.I.); marzafeir31@gmail.com (M.Z.-C.); kellybio4@hotmail.com (K.D.); 5 Faculty of Pediatrics, Pirogov Russian National Research Medical University, Moscow 117513, Russia; aleks80975@gmail.com; 6 Research and Clinical Center for Vision Restoration, Moscow 119021, Russia; dr.yaremenko@gmail.com; 7 Harvard Medical School, Brigham and Women’s Hospital, Boston, MA 02115, USA

**Keywords:** X-linked retinitis pigmentosa, gene therapy, RPGR gene, PR2 gene, vision restoration

## Abstract

X-linked retinitis pigmentosa (XLRP) is a severe inherited retinal degenerative disease characterized by progressive loss of photoreceptors and retinal pigment epithelium, leading to blindness. Predominantly affecting males due to mutations in the RPGR gene, XLRP currently lacks effective treatments beyond supportive care. Gene therapy has emerged as a promising approach to restore photoreceptor function by delivering functional copies of the RPGR gene. Recent clinical trials using AAV vectors, such as AAV5-RPGR and AGTC-501, have demonstrated encouraging results, including improvements in retinal sensitivity and visual function. While early successes like LUXTURNA have set the precedent for gene therapy in retinal diseases, adapting these strategies to XLRP presents unique challenges due to the complexity of RPGR mutations and the need for efficient photoreceptor targeting. Advances in vector design, including the use of optimized AAV serotypes with enhanced tropism for photoreceptors and specific promoters, have significantly improved gene delivery. Despite setbacks in some studies, ongoing research and clinical trials continue to refine these therapies, offering hope for patients affected by XLRP. This review explores the etiology and pathophysiology of XLRP, evaluates current treatment challenges, highlights recent clinical advances in gene therapy, and discusses future perspectives for bringing these therapies into clinical practice.

## 1. Introduction

X-linked retinitis pigmentosa (XLRP) is one of the most severe forms of inherited retinal degeneration, predominantly affecting males due to its X-linked inheritance pattern and leading to blindness [1]. In XLRP, night blindness typically presents in the first decade of life, followed by progressive loss of peripheral vision and eventually central vision [1]. Affected males often reach legal blindness at a median age of 45 years, compared to a median age of 70 years for retinitis pigmentosa (RP) in general [1,2]. This condition is marked by a gradual loss of photoreceptor cells, initially impacting night vision and peripheral vision, and often culminating in complete blindness by the age of 40–50 years [3]. XLRP accounts for about 10–15% of all retinitis pigmentosa cases and is primarily associated with mutations in the RPGR (retinitis pigmentosa GTPase regulator) and RP2 (retinitis pigmentosa 2, activator of ARL3 GTPase) genes, which play a fundamental role in maintaining photoreceptor structure and function [4,5,6,7]. Given its early onset and progressive nature, XLRP significantly impairs the quality of life and daily functioning of affected individuals, underscoring the urgent need for effective therapeutic options [3].

The importance of developing treatments for XLRP cannot be overstated. Currently, no cure exists, and management options are limited to supportive measures such as visual aids and vitamin supplementation, which do little to halt the disease’s progression [3,8,9]. As vision loss is often irreversible, delaying or preventing retinal degeneration in XLRP could profoundly impact the lives of affected individuals, allowing them to maintain functional vision for a longer period. For many patients, especially younger ones, a successful treatment could mean preserving their vision into adulthood, vastly enhancing their independence and quality of life [9].

Gene therapy has emerged as a beacon of hope in addressing the underlying causes of XLRP, rather than simply alleviating symptoms. By targeting the genetic mutation responsible for the disorder, gene therapy aims to restore normal function to the retinal cells, potentially stopping or even reversing vision loss [2,10,11]. Initial trials in other inherited retinal diseases, such as Leber congenital amaurosis (LCA), have shown that gene replacement therapies can be safe and effective, paving the way for similar strategies in XLRP [12]. Specifically, adeno-associated virus (AAV) vectors are being investigated to deliver functional copies of the RPGR gene directly into the retinal cells, enabling them to produce the essential proteins required for cell survival and function [11,13,14].

In addition to gene replacement, emerging genome-editing technologies, such as CRISPR/Cas9, hold the potential for correcting specific mutations within the RPGR gene itself [15]. Such approaches promise to offer a more precise and durable correction of the genetic defect, potentially leading to lasting therapeutic effects after a single treatment. However, achieving clinical success with gene therapy for XLRP presents several challenges, including the high variability in RPGR mutations, the need for targeted delivery to photoreceptors, and ensuring long-term safety and efficacy.

This review examines the latest clinical advances in gene therapy for XLRP, focusing on recent clinical trial outcomes, novel techniques, and translational challenges. We aim to provide a comprehensive overview of the current state of gene therapy research for XLRP, discussing both the successes and limitations encountered thus far.

## 2. Etiology and Pathophysiology of XLRP

**Figure 1:**
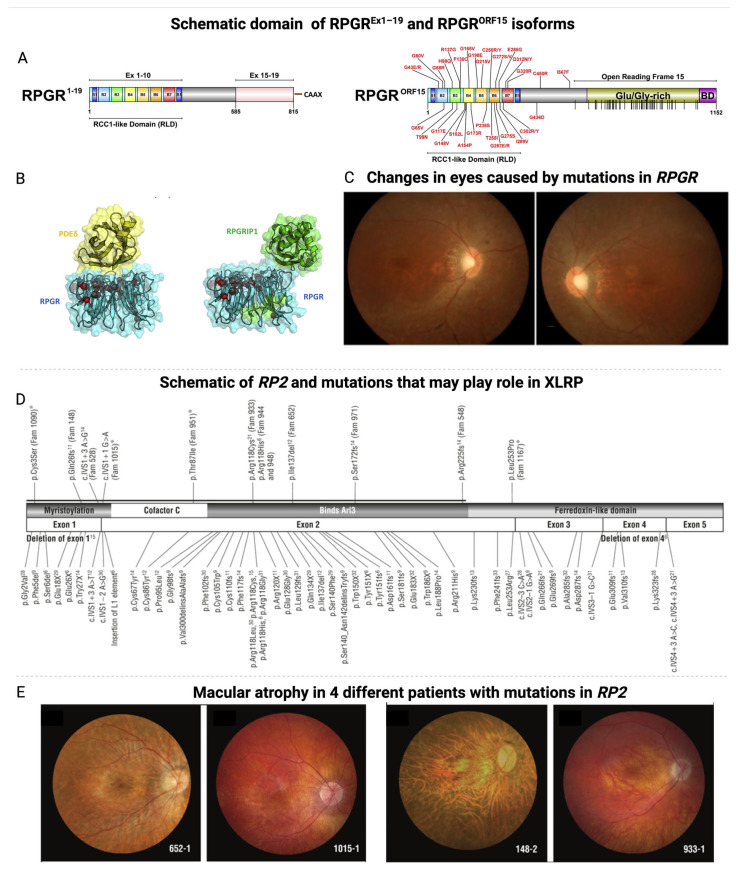
Domain schematics of major RPGR protein isoforms [23]. (**A**) The domain architectures of the two main RPGR isoforms—constitutive RPGR^Ex1−19^ and RPGR^ORF15^—are illustrated to scale. Both isoforms feature the beta-propeller RCC1-like domain (RLD), composed of seven blades (B1 to B7) encoded by exons 1–10. In RPGR^Ex1−19^, a C-terminal isoprenylation site (CAAX) is present. The RPGR^ORF15^ isoform contains a Glutamate/Glycine-rich domain and a Basic Domain (BD) within Open Reading Frame 15 (ORF15). Known disease-causing missense mutations are labeled, and 52 nonsense mutations within ORF15 are indicated by vertical lines on the schematic. (**B**) The crystal structure of the RPGR RLD (blue) in complex with PDE δ (yellow) is displayed using PyMol. A transparent surface representation highlights the locations of known missense mutations, shown as red spheres (alpha carbon atoms only). The interaction sites for PDE δ and RPGRIP1 on the RPGR surface partially overlap. Reproduced under terms of the CC-BY license [23]. 2015, Megaw et al., published by Elsevier Ltd. (**C**) A family with a novel nonsense mutation c.2833G>T (p.E945X) in the ORF15 region of the RPGR gene. Fundus photographs of the 30-year-old proband show disc pallor, attenuated blood vessels, and tapetal-like fundus changes. Reproduced under terms of the CC-BY license [4]. 2019, Zhang et al., published by BMC. (**D**) Illustration of previously identified and novel mutations in the RP2 gene. “Fam” denotes the family identifier, and novel mutations are indicated with an asterisk. (**E**) Macular atrophy observed in four different patients with *RP2* mutations. Reproduced under terms of the CC-BY license [24]. 2010, Jayasundera et al., published by American Medical Association.

RP is a genetic disease characterized by progressive degeneration of retinal pigment epithelium (RPE) and photoreceptors [16]. This disease is a heterogeneous collection of inherited retinal diseases that involves progressive retinal degeneration (~2 million people globally) [17,18]. RP can be syndromic or non-syndromic. Syndromic RP also affects other organs and tissues, whereas non-syndromic RP affects only the retina. In this review, we will focus on non-syndromic RP. More than 60 genes have been linked with non–syndromic RP, and they can be inherited in autosomal dominant, recessive, or X-linked recessive ways [10]. Rarer patterns like mitochondrial mutations, digenic diallelic inheritance, or incomplete penetrance have also been documented [16]. The severity of the symptoms and the onset of the disease vary depending on the mode of inheritance, with the autosomal ones manifesting later in life with milder phenotypes, while the X-linked ones start early and with more severe phenotypes. Variation in phenotypes in RPGR contributes to multiple disease patterns, including rod-cone dystrophy (70%), cone-rod dystrophy (6–23%), and cone dystrophy (7%) [19]. Features of RP include loss of visual acuity, progressive loss of visual field (VF), attenuation of retinal vessels, and the presence of bone-spicule deposits in the fundus, along with reduced or nondetectable a-waves and b-waves on electroretinogram examination. Most individuals with RP begin to experience night blindness during adolescence. This can be followed by a progressive loss of mid-peripheral visual fields in adulthood and eventually central vision loss, typically resulting in blindness by their fifties [18]. X-linked retinitis pigmentosa is a severe form of RP characterized by accelerated and advanced visual impairment. Pathogenic variants associated with XLRP affect predominately male individuals. Female carriers of a disease-causing variant sometimes can be affected clinically, and, in these cases, typically present with a milder phenotype than male patients, potentially due in part to random or skewed X chromosome inactivation [20,21,22]. The most common causes of XLRP are pathogenic variants in two genes, retinitis pigmentosa guanosine triphosphatase regulator (RPGR) and retinitis pigmentosa 2 (RP2) (Figure 1A–E).

In addition, several gene loci have been proposed for this condition, including RP3, RP6, RP23, RP24, and RP34 [25,26]. Mutations in the two major genes, RP2 and RP3, or the RPGR, have been studied more extensively than others [10]. However, diagnosing between RP2 and RPGR-related XLRP is particularly challenging, as there are no ocular measurements that are exclusively genotype-specific [20]. For example, a tapetal-like reflex may be present in both patients and carriers of either RPGR or RP2 mutations, complicating the differential diagnosis [20,27].

**Figure 2:**
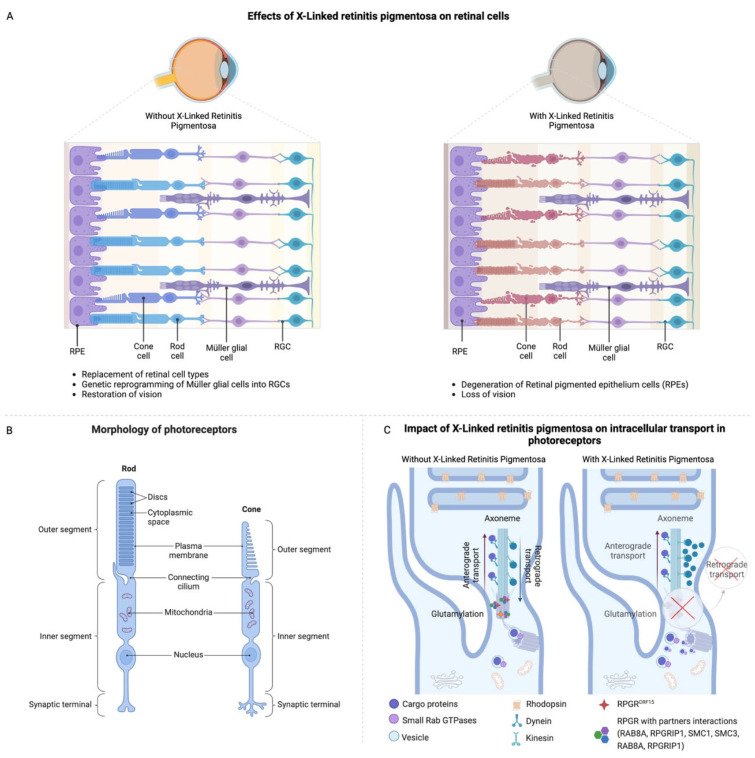
The pathophysiology and cellular impact of X-linked retinitis pigmentosa on retinal cells and photoreceptor function. (**A**) Effects of X-linked retinitis pigmentosa on retinal cells. Normal retinal cell architecture without X-linked retinitis pigmentosa, showing healthy cone and rod cells, Müller glial cells, and RPE. Restoration of vision strategies includes reprogramming Müller glial cells into retinal ganglion cells (RGCs) (**left**). Degeneration of RPE, cone, and rod cells, leading to disrupted retinal structure and vision loss with X-linked retinitis pigmentosa (**right**). (**B**) Morphology of photoreceptors [14]. (**C**) Intracellular transport in photoreceptors. Normal axonal transport, with efficient anterograde (cargo delivery to outer segments) and retrograde (recycling back to the cell body) transport (**left**). Mutations in RPGR protein impair transport pathways, disrupting retrograde transport but not anterograde. This leads to photoreceptor dysfunction [14,40].

The RPGR gene is located on the X chromosome, and it has a length of around 170 kbs, which forms up to 22 exons [28,29]. Among the isoforms, the RPGR gene has three predominant isoforms that can be found in the retina: the RPGR component encoded by exons 1–19 (RPGR1−19) is expressed in the transition zone in the majority of ciliated tissues, and specifically in the transition segment of ciliated cells, RPGR^ORF15^, the main isoform expressed in photoreceptor cells and playing a leading role in the function of photoreceptors, and RPGRskip14/15, where exons 14 and 15 are essentially skipped, a form that has not yet been studied in depth [30]. RPGR1−19 and RPGR^ORF15^ share the first 14 exons, with ORF15 extending into RPGR intron 15 and encoding a glutamic-acid- and glycine-rich domain. RPGR^ORF15^ is a variant hotspot, accounting for approximately 60–80% of XLRP cases, with the majority of variants causing frameshift or a premature termination codon (PTC) [3]. RPGR^ORF15^ is located in the photoreceptor connecting cilium (CC), a structure that links and acts as a gateway for protein trafficking between the photoreceptor biosynthetic inner segment (IS) and the light-sensing outer segment (OS). The transport of proteins, including the opsins, through the CC is tightly regulated, and its disturbance is implicated in many IRDs, including RPGR-linked XLRP [31]. Although male patients are primarily affected by RPGR-associated XLRP because of an X-linked recessive pattern of inheritance, female patients can still be carriers and generally exhibit mild symptoms or severe degeneration (male pattern) [10,17,18]. In the absence of RPGR function, ciliary gating appears compromised, and proteins may move more freely between compartments, disrupting cell homeostasis and consequently leading to cell death [32]. The failure of RPGR can lead to a biochemical and morphological retinal abnormality, resulting in progressive loss of vision and degeneration of the retina. As a result of mutations in the RPGR gene, proteins cannot be synthesized and transported into the ciliary space, resulting in an insufficient production or accumulation of defective proteins in the rod discs. In finer detail, in the absence of adequate intracellular transport, photoreceptors are unable to convert light into electrical signals, resulting in vision loss. A dysfunctional RPGR increases rod susceptibility to oxidative stress by reducing their ability to handle reactive oxygen species (ROS). As a result of ROS exposure, cells suffer damage to membranes, mitochondria, and lysosomes, which ultimately leads to cell death through apoptosis. Stress from the above dysfunctions activates microglial cells, resulting in the release of inflammatory mediators and cytokines. As a result of this inflammatory reaction, retinal degradation and cell death are accelerated (Figure 2A–C) [33].

RP2 variants are responsible for approximately 5% to 20% of XLRP [27,34]. The underlying mechanism of RP2-associated retinal degeneration in humans remains poorly understood, and existing animal models of RP2-linked XLRP do not fully replicate the severe phenotype observed in patients [35]. While these models, such as the RP2-knockout mice, offer valuable insights into the disease’s progression, they do not completely mimic the complex pathology seen in human cases, including the extent of cone degeneration and visual impairment [36,37].

The RP2 gene, located on Xp11.23, functions as a GTPase-activating protein (GAP) for the small GTPase ARL3 and is regulated by its guanine nucleotide exchange factor (GEF) ARL13B [35]. While ARL13B is localized to the ciliary axoneme, RP2 and ARL3 primarily reside at the basal body and centriole at the base of photoreceptors. Together, ARL3, its effectors UNC119 and PDE δ, and RP2 are thought to play a crucial role in trafficking lipidated proteins such as transducin, GRK1, and PDE6 into the photoreceptor outer segment, a process essential for visual signal transduction [35]. So, RP2 encodes a protein that plays a crucial role in retinal health, specifically in the trafficking of myristoylated and prenylated proteins to the photoreceptor cilia, a structure essential for retinal function. The RP2 protein is structurally similar to cofactor C, which is involved in the folding of β-tubulin, a key component of the retinal microtubule network, further highlighting its importance in cellular maintenance and protein stability within photoreceptor cells [38]. Mutations in the RP2 gene can result in various degrees of retinal degeneration, but there remains a lack of clear genotype–phenotype correlations [39]. These discrepancies highlight the need for more refined models that better capture the human disease phenotype for the development of effective treatments. While RP2 mutations typically cause a progressive loss of vision, the severity of the disease varies among individuals [20]. Clinical features of RP2-associated XLRP include night blindness, reduced visual acuity, and peripheral vision loss, which often begins in childhood and progresses to severe vision impairment by early adulthood. Some mutations in RP2 have been linked to more severe forms of the disease, although identifying specific mutations that correlate with faster progression remains an area of active investigation [35]. The differential diagnosis between RP2 and other XLRP-related mutations, such as those in the RPGR gene, is particularly challenging. This is because no ocular measures, such as the tapetal-like reflex, are specific to one genotype or another. The overlap of symptoms further complicates the ability to predict disease severity based solely on the genetic mutation. Given its central role in retinal cell function, the RP2 gene is a target of ongoing research into gene therapies for XLRP. However, as of now, no targeted treatments have been developed for RP2-related XLRP. Understanding the detailed molecular mechanisms by which RP2 mutations lead to retinal degeneration is essential for the development of effective therapeutic strategies.

## 3. The Current Treatments of XLRP and Their Challenges

Current treatments for XLRP primarily address the symptoms and complications arising from the progressive nature of the disease, focusing on preserving remaining vision and maintaining quality of life [41]. Management of XLRP involves a structured approach to monitoring and treating the common complications associated with retinal degeneration, particularly cystoid macular edema (CME) and cataract formation [42]. Patients are regularly monitored for the development of CME and cataracts, with treatment tailored to slow the visual decline and optimize the functional vision that remains. The emotional toll of progressive vision loss is significant, so psychological support is often integrated into care plans, helping patients cope with the social and personal challenges of diminishing sight [3,18,43].

Pharmacologic interventions, especially for managing CME, are a key component of XLRP treatment. Intravitreal injections of anti-VEGF agents, such as bevacizumab, aim to reduce fluid accumulation in the macula, where edema can accelerate central vision loss [9]. Anti-VEGF therapy, targeting abnormal vascular permeability, has been helpful for many patients, though responses to this treatment can vary, often necessitating ongoing or repeated injections to achieve desired results. When anti-VEGF alone is insufficient, corticosteroids are sometimes administered intravitreally to combat inflammation. Corticosteroids like triamcinolone work by suppressing retinal inflammation, which can provide further relief from CME [9,42]. However, corticosteroid use requires careful balancing, as extended use can increase the risk of complications such as elevated intraocular pressure and additional cataract formation.

For those with cataracts resulting from XLRP, surgical intervention remains a beneficial yet complex choice. Cataract surgery, typically performed via phacoemulsification with intraocular lens placement, can significantly improve clarity of sight for many patients, although it must be carefully timed to avoid exacerbating retinal stress. This surgical approach, while effective for clearing visual obstructions caused by cataracts, cannot prevent the underlying progression of retinal degeneration, underscoring the need for emerging therapies that target XLRP at a genetic or cellular level.

In XLRP treatment, vitaminotherapy involving Vitamin A, Omega-3 fatty acids (particularly docosahexaenoic acid), lutein, and zeaxanthin has also been explored as a potential strategy for enhancing retinal health and slowing disease progression [10,44]. These compounds are believed to provide antioxidant and anti-inflammatory benefits, which may help preserve retinal function. Studies have indicated that Vitamin A (specifically retinyl palmitate) may slow the decline in retinal function, while Omega-3 fatty acids can enhance the effect by protecting against inflammation and preserving retinal cells [10]. Lutein and zeaxanthin, potent antioxidants, may further support retinal integrity by reducing oxidative damage [10]. However, high doses of Vitamin A require caution due to risks such as liver toxicity, and these supplements are not yet established as standard care due to limited long-term data and the need for precise dosing to avoid adverse effects [10,45,46]. Moreover, while promising, this approach is not yet a standard treatment due to concerns about safety, particularly with high-dose Vitamin A, and the need for more extensive clinical validation [10].

**Figure 3:**
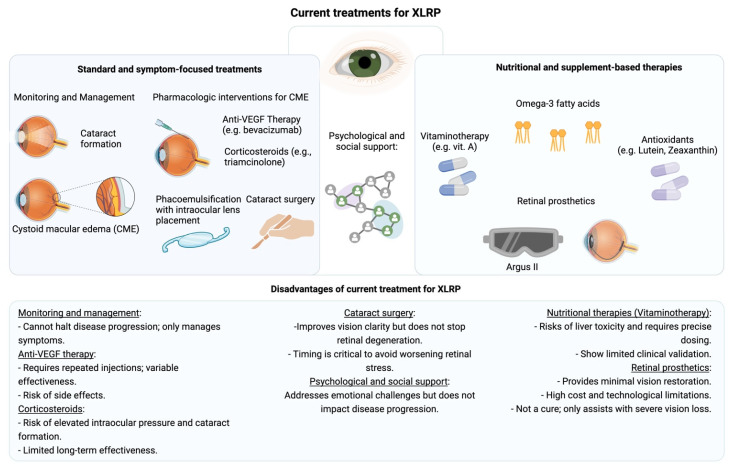
The current treatments for XLRP and their associated disadvantages.

Additionally, in recent years, innovative therapies, such as retinal prosthetics, have provided some hope for functional improvement in vision. Devices like the Argus II retinal prosthesis offer limited visual restoration by enabling patients to perceive basic light patterns and shapes [1,47]. The technology, while not a cure, represents a meaningful advancement in assistive options, allowing for some level of independence in daily activities for those with severe vision loss. However, the limitations of current technology mean that the level of vision provided remains minimal, and the treatment landscape remains centered on symptomatic management rather than a direct resolution of the genetic causes of XLRP (Figure 3) [41].

As a whole, while current XLRP treatments provide relief and improve quality of life by addressing symptoms and complications, they do not alter the underlying disease trajectory. Future advances in gene therapy and neuroprotective strategies hold promise for shifting treatment from symptomatic care to potentially curative approaches. Until then, XLRP treatment is focused on preserving visual function and supporting patients in managing the challenges associated with gradual vision loss.

## 4. Gene Treatment of XLRP

Gene therapy represents a promising avenue for the treatment of XLRP. It makes it possible to replace or correct the defective RPGR and RP2 genes, thereby restoring the normal function of the photoreceptors and slowing or halting disease progression [2,10,37,43]. Several gene therapy strategies for XLRP are currently under investigation, with most relying on AAV vectors to deliver the healthy RPGR gene directly to the retina or using CRISP/Cas9 technology [15]. AAV vectors are non-pathogenic, capable of infecting an organism but never causing disease because their pathogenicity has been neutralized. They are mostly used in gene therapies because they can be modified to target specific cell types like retinal pigment epithelium and photoreceptors [10].

### 4.1. Vectors Utilized in Ocular Gene Therapy

Recent breakthroughs in pre-clinical trials are offering promising advances in XLRP care, targeting genetic mutations that lead to progressive vision loss. Central to these developments is the use of AAV vectors, which have shown remarkable potential in safely delivering functional genes to retinal cells, highlighted by early-phase clinical trials.

The AAV variants differ in their capsid components and thus exhibit distinct cellular tropism, transduction efficiency, and immunogenicity [48]. About a dozen different naturally occurring AAV serotypes have been identified in primates with more than 100 variants. Naturally occurring AAV1, AAV2, AAV4, AAV5, AAV6, AAV7, AAV8, and AAV9 all display tropism for retinal tissue [10,48,49]. All of these serotypes efficiently transduce RPE, especially following SR delivery; however, the transduction of photoreceptors and other cell types within the retina differs among the various serotypes. The naturally occurring serotypes AAV2, AAV5, and AAV8 have been most widely studied for use as ocular gene therapy vectors [50]. These three vectors work most efficiently via an SR delivery route because transduction of retinal cells following injection is limited by the ILM as well as pre-existing neutralizing antibodies. For example, depending on geographic region, 30% to 60% of humans have neutralizing antibodies (nAbs) to AAV2; these nAbs have the potential to severely limit cellular transduction and transgene protein production [10]. Many recombinant vectors in use today are composed of components of the AAV2 serotype combined with the capsids of AAV1 (AAV2/1), AAV4 (AAV2/4), AAV5 (AAV2/5), AAV6 (AAV2/6), AAV7 (AAV2/7), AAV8 (AAV2/8), and AAV9 (AAV2/9) [10]. In addition to attaining the desired cellular tropism and enhancing transduction efficiency, pseudotyping can also circumvent pre-existing immunity to certain AAV serotypes that could affect the transduction efficacy and potentially the safety of an ocular gene therapy. All AAV pseudotypes can transduce RPE, with AAV2/1, AAV2/4, and AAV2/6 being the most specific. In contrast, only AAV2/5, AAV2/7, AAV2/8, and AAV2/9 efficiently transduce photoreceptors, with AAV2/8 and AAV2/9 being the most efficient across a number of species. The pseudotypes AAV2/5, AAV2/8, and AAV2/9 transduce the highest percentage of cone photoreceptors [10]. Recombinant AAV (rAAV) vectors have been designed for gene therapy by replacing the rep and cap genes with the transgene cassette of interest (e.g., RPE65 in the case of Luxturna and anti-VEGF protein in the case of RGX-314 (REGENXBIO) and ADVM-022 (Adverum Biotechnologies)). The rep and cap ORFs are then provided during the AAV manufacturing process via helper plasmids. rAAV2, the first serotype to be successfully used for gene therapy, efficiently transduces RPE cells following SR delivery (e.g., LUXTURNA). For gene therapies targeting photoreceptor cells, AAV8 is the most efficient option, followed by AAV2/5 and AAV5. AAV2 is less efficient unless modified but still useful in some contexts like retinal pigment epithelial cell targeting [10,48,49].

### 4.2. Immune Challenges and Side Effects of AAV-Based Gene Therapies

AAV-based gene therapies have emerged as a promising approach for treating inherited retinal diseases. However, the immune response elicited by these therapies presents significant challenges that can impact their efficacy and safety. The AAV capsid, transgene, and promoter can all serve as immunogenic triggers, eliciting both innate and adaptive immune responses [51,52,53,54].

Upon administration, AAV vectors can activate the innate immune system. This activation is mediated through pattern recognition receptors such as Toll-like receptors (TLRs), which recognize viral components and initiate inflammatory responses. For instance, TLR9 has been implicated in detecting AAV’s DNA, leading to the production of pro-inflammatory cytokines. This innate response can result in transient inflammation and may influence the transduction efficiency of the vector [51,52,53,54].

The adaptive immune system also can mount a response against AAV vectors through both humoral and cellular mechanisms. Pre-existing neutralizing antibodies against AAV, resulting from natural infections, can impede vector delivery by binding to the viral capsid and preventing cellular entry. Additionally, the transgene product may be recognized as foreign, especially in cases where patients lack the endogenous protein due to genetic mutations. This recognition can lead to the activation of cytotoxic T lymphocytes that target and destroy transduced cells, thereby diminishing therapeutic efficacy. Moreover, the eye is considered an immune-privileged organ, characterized by mechanisms that suppress immune responses to maintain visual function. These mechanisms include the blood–retinal barrier and the expression of immunomodulatory factors that promote immune tolerance. However, in the context of IRDs, the degenerative environment can compromise this privilege. Inflammation associated with disease progression may disrupt the blood–retinal barrier, allowing immune cells to infiltrate retinal tissues. Consequently, AAV-mediated gene therapies in such compromised environments may encounter heightened immune responses, leading to adverse effects like gene therapy-associated uveitis and reduced transgene expression [51,52,53,54].

To enhance the success of AAV-based gene therapies, several strategies are being explored to mitigate immune responses. First and foremost, modifying the AAV capsid to evade recognition by nAbs and reduce the activation of pattern recognition receptors is a critical strategy to decrease immunogenicity. For example, the development of novel serotypes or engineered capsids with reduced affinity for pre-existing antibodies can significantly improve vector delivery and therapeutic outcomes [51,52,53,54]. Additionally, determining the minimal effective dose of AAV vectors is essential, as it can reduce the antigenic load and subsequent immune activation while still achieving the desired therapeutic benefit. Another effective method for reducing immune responses involves administering immunosuppressive agents in conjunction with gene therapy. These agents can help dampen both innate and adaptive immune responses, enhancing the therapy’s efficacy. However, this approach must be carefully managed to avoid the potential risks associated with systemic immunosuppression, such as increased susceptibility to infections. The choice of delivery route also plays a crucial role in mitigating immune responses and optimizing transduction efficiency. Selecting subretinal delivery instead of intravitreal injections can take advantage of the localized immune privilege within the subretinal space, thereby minimizing immune activation [51,52].

A comprehensive understanding of the complexities of immune responses to AAV-based gene therapies is fundamental for developing effective and long-lasting treatments for IRDs. Continued research into these strategies will help refine therapies to minimize immunogenicity and maximize patient outcomes [55,56,57,58,59,60].

AAV-RPGR gene therapies, while promising, are not without risks [56,57,59]. Adverse effects (AEs) have been reported in all trials conducted to date. However, the lack of systematic and uniform reporting, combined with the use of corticosteroids to suppress inflammation, complicates the formal recording of the severity and frequency of these events. Most AEs are mild and transient, often stemming from the nature and method of administration. Subretinal or intravitreal injections, for instance, are inherently invasive procedures and can lead to subconjunctival hemorrhages, irritation, pain, blurred vision, diplopia, and fluctuations in intraocular pressure [57]. Subretinal injections, in particular, require vitrectomy, which further increases the risk of cataract formation. More severe adverse effects—accounting for approximately 8% of total AEs—have been reported within the first 30 days of treatment and included irreversible decreases in visual acuity [57]. These severe effects encompassed macular and perifoveal atrophy, retinal thinning, loss of outer retinal architecture, and steroid-induced glaucoma. Ocular inflammation, predominantly observed with higher doses of viral vectors (above 1.5 × 10^11^ viral genomes), was generally manageable with immunosuppression and corticosteroids [57].

### 4.3. Gene Therapy of XLRP in Pre-Clinical Trials

Pre-clinical gene therapy trials for XLRP focus on developing therapeutic strategies, including gene replacement and gene editing of RPGR or RP2 genes directly to affected retinal cells, with the goal of preserving or restoring vision [15]. These studies involve testing various serotypes of AAV vectors in vitro and in vivo to determine the most effective vectors for precise, sustained gene delivery to retinal cells.

Gene replacement and gene editing for XLRP have made significant strides, with several studies demonstrating promising preclinical results using AAV vectors [10]. Choosing the most effective AAV serotypes for targeted delivery is crucial for advancing gene therapy as a viable treatment for XLRP.

One of the first positive outcomes was achieved with the AAV2/5-hIRPB-hRPGR vector in vivo of XLRP, which is similar to the human condition [61]. After subretinal injections, the dogs’ retinas showed not only preservation of photoreceptor function and structure but also reversal of secondary retinal remodeling in the outer plexiform layer (OPL) and inner retinal layers. This groundbreaking study marked a significant step forward in gene therapy for retinal diseases [61]. Later, the use of AAV8 and AAV9 vectors with RPGR transgenes in mice produced even more robust results, with a clear dose–response relationship. For example, in eyes treated with AAV9-mRPGR, a significantly thicker outer nuclear layer was maintained compared to control eyes, as evidenced by optical coherence tomography imaging at 18 months post-injection. Treated eyes also exhibited greater overall retinal thickness across a ∼1.0 mm^2^ field of view, except in areas near the optic nerve head. Immunofluorescence analyses revealed that RPGR expression extended across approximately half of the retinal cross-sections in vector-treated eyes. This expression correlated with the preservation of seven to ten rows of photoreceptors, compared to only four to six rows in untreated controls. Moreover, vector-treated retinas maintained outer nuclear layer thicknesses ranging from 31.7 to 43.5 µm, while control retinas ranged from 19.0 to 28.3 µm. The therapeutic effect was even more pronounced at 24 months post-injection in mice receiving a higher dose of AAV8-mRPGR. In these cases, 6 to 8 rows of photoreceptors were preserved in inferior retinal regions expressing RPGR, while control eyes retained only one to three rows, with nearly complete outer nuclear layer loss in some superior regions. These results highlight the potential of AAV-mediated RPGR gene therapy to effectively preserve photoreceptor integrity and retinal structure over extended periods, providing a strong foundation for its application as a therapeutic strategy for XLRP [37]. However, toxicity was observed at higher doses. Despite this, retinal function and structure remained well-preserved, with clinical improvements seen even after the onset of retinal degeneration [37]. This was further confirmed in a study combining the AAV2/5 vector with full-length human RPGR1^ORF15^ in vivo, where high doses did not induce toxicity, marking a significant improvement over the mouse model and suggesting that AAV2/5 might be the most effective vector for gene delivery in XLRP therapies [36]. In a bid to optimize gene delivery further, two different promoters were tested—interphotoreceptor retinoid-binding protein (IRBP) and G protein-coupled receptor kinase 1 (GRK1). The IRBP promoter drives transgene expression in both rods and cones, making it suitable for therapies targeting a broad range of photoreceptors [62]. In contrast, the GRK1 promoter is more specific to rods, providing targeted expression for rod-associated functions or diseases [63].

**Figure 4:**
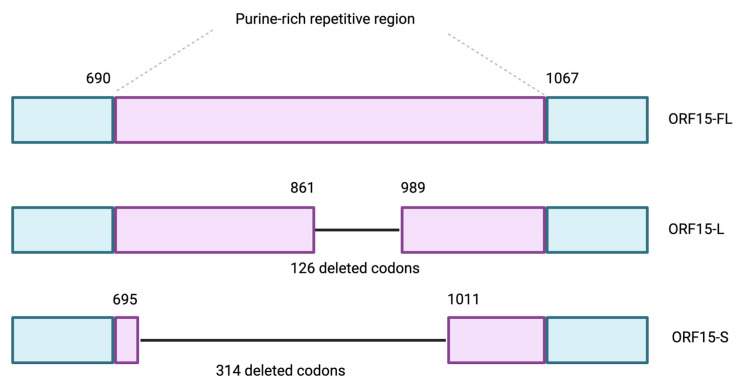
Diagrams of the wild-type RPGR *ORF15-FL* coding region and AAV-delivered cDNA coding regions of two shortened forms of RPGR *ORF15-L* and *ORF15-S* [64].

The GRK1 promoter proved more effective for driving expression of the gene specifically in photoreceptors, enhancing the efficacy of the gene therapy [43]. Further advancements involved targeting the RPGR^ORF15^ region, a purine-rich area of the gene prone to mutations. For example, there were two shortened forms of RPGR tested: one retaining two-thirds of the purine-rich region and one with most of it deleted (Figure 4). The results showed that the longer form of the gene provided better stability and function than the truncated variant, which lacked efficiency [64].

**Figure 5:**
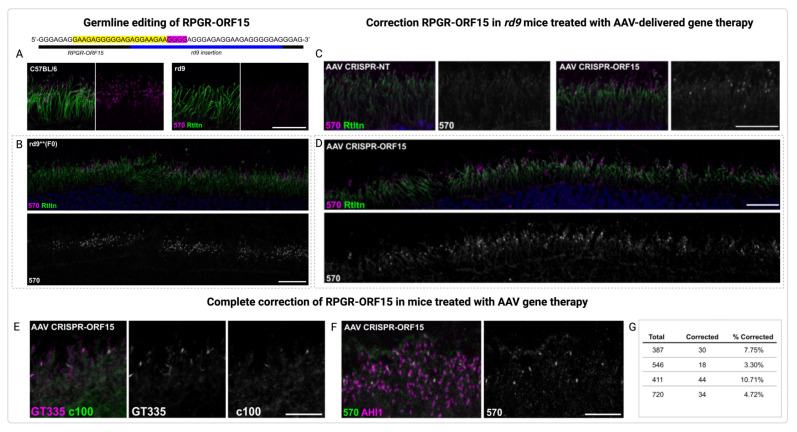
*In vivo* restoration of RPGR expression via CRISPR/Cas9 gene editing and AAV-RPGR gene therapy. (**A**–**C**) Germline editing of RPGR-ORF15 [66]. (**A**) Schematic illustrating the target region of the ORF15 in rd9 mice. The sgRNA complementary sequence is marked in yellow, while the PAM sequence is indicated in magenta. (**B**) Confocal microscopy images displaying RPGR-ORF15 protein expression localized at the cilia in control (C57BL/6) retinas, detected using the ORF15-specific antibody RPGR-570. In rd9 retinas, RPGR-ORF15 expression is absent, with the ciliary rootlet visualized using Rootletin (Rtltn) staining. Below: Confocal images of a *rd9* founder mouse following CRISPR/Cas9-mediated gene editing. (**C**,**D**) Correction RPGR-ORF15 in *rd9* mice treated with AAV-delivered gene therapy. Confocal images of AAV-treated *rd9* retinal cross-sections showing successful gene editing. RPGR-ORF15 expression is observed exclusively in ORF15-targeted retinas (AAV CRISPR-ORF15), with no expression in non-targeted retinas (AAV CRISPR-NT). A subset of cells shows RPGR-570 localization at the connecting cilium, with the ciliary rootlet highlighted by Rootletin (Rtltn) staining. (**D**) A lower magnification view of panel (**C**) demonstrates the broad distribution of corrected cells across the retina. The lower panel also includes a desaturated 570 channel [66]. (**E**–**G**) Complete correction of RPGR-ORF15 in mice treated with AAV gene therapy. (**E**) Confocal images of CRISPR-targeted ORF15 (AAV CRISPR-ORF15) retinal cross-sections from *rd9* mice. Staining with additional RPGR-specific antibodies, c100 and GT335, confirms the expression of RPGR-ORF15 in treated retinas. (**F**) Staining with RPGR-570 and AHI1 in AAV CRISPR-ORF15 treated *rd9* retinal cross-sections shows that all connecting cilia are positive for AHI1, while a subset is also positive for RPGR-570, indicating successful correction of RPGR-ORF15 in these cells. Scale bars in A–F indicate 20 μm. (**G**) Quantification of RPGR-ORF15 rescue across four large fields in AAV CRISPR-ORF15 treated retinas. Reproduced under terms of the CC-BY license [66]. 2022, Gumerson, J. et al., published by Springer Nature.

To address the problems associated with the purine-rich region of ORF15 of the RPGR gene, extensive codon optimization efforts were conducted [65]. Another in vivo study investigated the restoration of RPGR expression using CRISPR/Cas9 gene editing to treat XLRP, a severe form of inherited retinal degeneration caused by mutations in the RPGR gene in fertilized *rd9* mouse embryos (Figure 5A–G) [66].

Corrected mice expressed full-length RPGR-ORF15 protein, localized properly in photoreceptors, and showed improved retinal health. Corrected mice expressed the full-length RPGR-ORF15 protein, which localized correctly in photoreceptors and was associated with significant improvements in retinal health and structure. Subretinal injections of AAV-delivered CRISPR/Cas9 components targeted RPGR mutations in the mature photoreceptors of *rd9* mice, restoring RPGR function in a subset of cells. This restoration not only improved retinal structure but also reduced opsin mislocalization [66].

Studies have shown that AAV-RPGR treatment positively impacts both rod and cone photoreceptors, though their responses to the therapy differ. Rod photoreceptors, which are typically affected earlier in XLRP, showed significant preservation of structure and function after AAV-RPGR treatment. Cone photoreceptors, which are critical for central and color vision, also demonstrated improved health and survival, though their restoration was less pronounced compared to rods. This differential response may reflect the distinct cellular demands and vulnerability of rods and cones in the disease context. Nevertheless, AAV-RPGR therapy effectively reduced photoreceptor loss and maintained retinal integrity over time, highlighting its therapeutic potential for long-term vision preservation. The therapeutic benefits were evident in the maintenance of outer nuclear layer thickness and photoreceptor organization, further emphasizing the promise of AAV-RPGR gene therapy in treating XLRP. So, gene editing restored RPGR function in a subset of cells, improving retinal structure and reducing opsin mislocalization [66].

**Figure 6:**
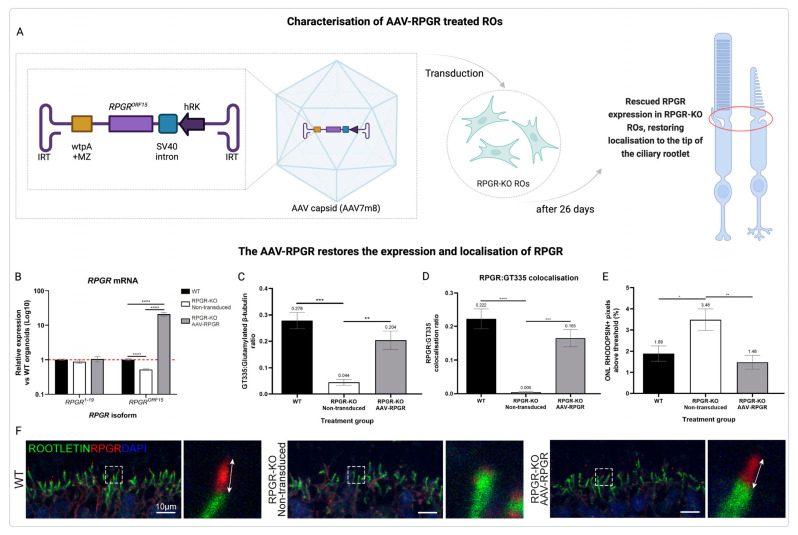
AAV-RPGR treatment restores RPGR expression, localization, and function in retinal organoids (ROs). (**A**) Characterization of AAV-RPGR treated ROs (red circle indicates the region of changes) [31]. (**B**) AAV-RPGR significantly increases RPGR-ORF15 expression, normalized to GAPDH and ACTIN, reaching WT levels [31]. Red dashed line demarcates normalised WT expression levels. (**C**) Quantification confirms higher RPGR glutamylation levels in treated ROs. (**D**) Quantification shows improved RPGR:GT335 co-localization in treated ROs. (**E**) Rhodopsin mislocalization in RPGR-KO ROs is reduced by AAV-RPGR, restoring proper distribution. (**F**) AAV-RPGR corrects RPGR localization to the apical tip of the ciliary rootlet in RPGR-KO ROs, as seen in WT ROs. (**F**) Mislocalization in untreated ROs is resolved, with correct localization highlighted by arrows. Scale details and nuclei stained with DAPI (blue) [31]. Main image scale bar = 10 μm, zoom image (white square) scale bar = 2 μm, White arrows represent the region of RPGR localisation. Reproduced under terms of the CC-BY license [31]. 2024, Sladen, P. et al., published by MDPI. * *p* < 0.05, ** *p* < 0.01, *** *p* < 0.001, **** *p* < 0.0001.

Another study investigated the efficacy of AAV-RPGR gene therapy in a human retinal organoid model of RPGR-associated XLRP (Figure 6A–F) [31]. Using CRISPR/Cas9-generated RPGR-knockout (KO) induced pluripotent stem cells (iPSCs), researchers differentiated ROs and treated them with an AAV vector delivering a clinical RPGR^ORF15^ construct. The results demonstrated that AAV-RPGR (vector AAV7m8) successfully restored RPGR mRNA and protein expression in RPGR-KO ROs. Importantly, RPGR localization to the connecting cilium of photoreceptors was achieved, closely resembling the wild-type (WT) organoids. Additionally, vector-derived RPGR exhibited glutamylation levels comparable to WT, a critical modification for proper photoreceptor function. Furthermore, mislocalized rhodopsin in RPGR-KO ROs was corrected, restoring proper trafficking within photoreceptor cells. This study underscores the potential of AAV-RPGR therapy to address the underlying molecular defects in XLRP. By restoring critical gene expression, cellular localization, and protein function, it provides a robust foundation for advancing gene therapy as a therapeutic approach for retinal degenerative diseases [31].

Recent studies demonstrated that the development of the AAV7m8 capsid represents a significant advancement in gene therapy for XLRP [58,67]. AAV7m8 is an engineered capsid that exhibits enhanced transduction efficiency in retinal cells, particularly photoreceptors, which are critical for restoring visual function. Studies have shown that AAV7m8 facilitates robust expression of the therapeutic RPGR-ORF15 transgene, leading to the proper localization of RPGR protein in photoreceptor cilia [58,67]. One notable advantage of AAV7m8 is its ability to transduce retinal cells via intravitreal injection, a less invasive delivery method compared to subretinal administration. Intravitreal delivery minimizes surgical risks while still achieving therapeutic levels of transgene expression in target cells. Additionally, the AAV7m8 capsid demonstrates reduced immunogenicity compared to other capsids, further enhancing its suitability for clinical applications [58,67].

So, the approach aimed to modify the coding sequence of the gene using synonymous codons to reduce the likelihood of mutational hotspots and improve gene stability and expression. In addition, codon optimization helped to bypass the high mutation rates and production of splice variants associated with the purine-rich sequence, thereby enhancing the functionality of the gene and its potential for therapeutic use in XLRP [65]. Thus, by modifying the RPGR gene sequence without altering the encoded amino acids, a more stable and efficient version of the gene, called coRPGR, was created. This optimized gene was injected into retinal models lacking functional RPGR, and the results showed full functionality of the protein at the cilium, with no adverse effects and clear therapeutic potential [65].

In parallel to therapy XLRP caused by mutations in the RPGR gene, some studies showed promising results for therapy XLRP caused by mutations in the RP2 gene [35,68]. For example, Mookherjee et al. aimed at developing gene therapy for XLRP, specifically targeting the RP2 mutation, using RP2-knockout [68]. In this study, early-onset cone dysfunction and progressive cone degeneration were observed, mimicking the vision impairment seen in XLRP patients. Using a self-complementary AAV8 vector carrying the human RP2 gene to mediate stable RP2 protein expression in mouse photoreceptors [68]. The results showed that the gene therapy preserved cone function over 18 months, restored cone viability, corrected mis-trafficking of opsins, and restored critical enzyme expression. Although retinal toxicity was observed at high doses, the wide range of effective doses and long-lasting therapeutic effects suggest this therapy holds promise for clinical application) [68]. In another study, gene-edited isogenic RP2 knockout iPSCs and RP2 patient-derived iPSCs were used to create 3D ROs as a human model for XLRP [35]. The RP2 KO and patient-derived organoids showed significant rod photoreceptor cell death by day 150, with thinning of the outer nuclear layer (ONL) by day 180. Gene augmentation using an AAV vector carrying the human RP2 gene rescued this degeneration, preventing ONL thinning and restoring rhodopsin expression. These findings suggest that 3D ROs can effectively model photoreceptor degeneration and serve as a platform for testing gene therapies for XLRP [35].

These studies underscore significant progress in gene therapy for XLRP, particularly in restoring proper opsin localization to the photoreceptor outer segments, for example, the photoreceptor’s outer nuclear layer, and mitigating photoreceptor degeneration through AAV-RPGR that is critical for maintaining photoreceptor function. Despite these advancements, challenges remain, including optimizing vector delivery systems for efficient and targeted transduction, as well as minimizing toxicity associated with higher doses. Encouragingly, the consistent success in preclinical trials, including the restoration of RPGR function and correction of opsin mislocalization, brings renewed hope for the development of safe and effective treatments that could transform the management of XLRP in the near future.

## 5. Recent Clinical Advances in Gene Therapy for XLRP Care

Based on clinical trial data for LUXTURNA, which targets RPE65 mutations, it is reasonable to expect that future developments using AAV2 capsid technology targeting RPGR gene mutations may achieve similar success in clinical phases [69,70]. However, it is important to note that RPE65 is expressed in both RPE and photoreceptor cells, which may contribute to the high efficacy observed in LUXTURNA. In contrast, RPGR is expressed primarily in photoreceptors, suggesting different therapeutic challenges and outcomes for AGTC-501, AAV5- RPGR, and BIIB112 [71,72,73,74,75,76].

AGTC-501 is an investigational gene therapy developed for the treatment of XLRP caused by mutations in the RPGR gene [71]. This therapy utilizes a recombinant AAV2 vector with tyrosine-to-phenylalanine mutations (rAAV2tYF). These mutations in the AAV2 capsid reduce phosphorylation and subsequent degradation by cellular proteasomes, enhancing vector stability and transduction efficiency. The vector expresses the human RPGR^ORF15^ gene under the control of the GRK1 promoter, ensuring targeted expression in photoreceptor cells and minimizing off-target effects in other retinal cell types. In clinical trials, AGTC-501 has demonstrated a promising efficacy and safety profile [71,73]. For example, visual function improvement was 63% of eyes treated with a high dose of AGTC-501. Moreover, patients exhibited an improvement of greater than 7 decibels (dB) in at least five loci over 12 months [71]. These results indicate significant improvements in retinal sensitivity and suggest that AGTC-501 can enhance visual function in patients with XLRP and can be used for therapy for XLRP caused mutations in the PR2 gene.

In the development of AAV5-RPGR, a proprietary splicing-based riboswitch was designed for precise control of therapeutic gene expression using oral small molecule inducers [73]. This innovative technology uses synthetic riboswitches, a type of engineered RNA sequence, which can specifically and reversibly bind to small molecule inducers to control gene activity. The riboswitch platform of AAV5-RPGR operates via a mechanism where a synthetic RNA sequence, designed to function in mammalian cells, incorporates a small molecule-binding aptamer. This sequence acts as a switch: in the absence of the inducer, a premature stop codon prevents gene expression. When the inducer is present, it binds to the aptamer, triggering splicing that removes the stop codon, allowing the creation of stable mRNA and subsequent protein production. This system enables dose-dependent, reversible, and precise control of gene expression in targeted tissues. Moreover, the use of AAV2/5 capsids in AAV5-RPGR improves transduction efficiency to 12–20%, compared to 5–16.4% with AAV2 used in AGTC-501. The AAV5 capsid has an effective tropism for both photoreceptor cells and RPE cells when delivered subretinally [73]. This indicates the potential for AAV5-RPGR to deliver effective gene therapy for XLRP. In clinical trials, the therapy demonstrated a favorable safety profile. Approximately 96.9% of participants in the treatment groups reported AEs considered related to the surgical procedure rather than the gene therapy itself [73]. These AEs were transient, resolving without intervention, and included conjunctival hemorrhage, reduced visual acuity, and the presence of anterior chamber cells. Efficacy results from the trials were promising. Around 50% of patients treated with AAV5-RPGR exhibited significant improvements in visual function. Specifically, retinal sensitivity measurements at 12 months post-treatment showed a mean improvement of 0.95 decibels (dB) in the gene therapy group, compared to a mean decrease of −0.59 dB in the untreated group. Microperimetry assessments using the Macular Integrity Assessment (MAIA) device revealed a mean improvement of 5.6 dB in the treated eyes at 12 months, whereas the control group experienced a mean decrease of −1.2 dB [73]. Moreover, functional vision assessments conducted nine months after treatment indicated substantial enhancements in patients’ ability to perform visual tasks. Participants who received the gene therapy completed the assessments in an average of 16.4 s without errors, a significant improvement from their baseline performance of 61.7 s with two errors [73]. These findings suggest that AAV5-RPGR not only stabilizes but may also improve visual function in patients with XLRP. The therapy’s ability to target photoreceptor cells effectively, coupled with its encouraging safety and efficacy profiles, positions AAV5-RPGR as a promising candidate for treating XLRP. As of 2024, MeiraGTx is conducting two active Phase 3 trials (NCT04671433 and NCT04794101) and has one in recruitment (NCT05926583) (Table 1). These trials are expected to have sufficient sample sizes, avoiding the challenges faced in BIIB112’s Phase 3 trial.

BIIB112, also known as cotoretigene toliparvovec, is an investigational gene therapy developed for the treatment of XLRP caused by mutations in the RPGR gene [74]. This therapy employs an AAV8 vector to deliver a codon-optimized version of the human RPGR^ORF15^ gene [74,75]. In clinical trials, BIIB112 demonstrated a safety profile that requires careful consideration. Approximately 94% of participants experienced 35 treatment-emergent adverse events (TEAEs) associated with the study procedure. The most common adverse events were related to inflammation, reported in 67% of patients. Serious TEAEs occurred in 28% of participants and included: reduced visual acuity, noninfective retinitis, retinal detachment, and visual impairment. Regarding efficacy, the results from the clinical trials were modest, and despite few improvements in some patients, the therapy did not meet the primary endpoints in the Phase II/III XIRIUS study. The lack of significant difference between the treated and control groups may be attributed to several factors, including a high placebo response (22%), which reduced the observed treatment effect. Also, it is possible that this study showed variability in gene transduction efficiency and issues with sample size, patient selection, or outcome measures that may have impacted the results [74].

Thus, the challenges faced by BIIB112 emphasize the complexities involved in developing effective gene therapies for XLRP. The occurrence of serious adverse events suggests that the AAV8 vector or the delivery method may provoke immune responses or other unintended effects in the eye.

While BIIB112 has not yet achieved the desired clinical outcomes, the data obtained provide valuable insights for future research. Ongoing efforts aim to address the hurdles encountered in this program. Lessons learned from BIIB112 may contribute to the advancement of other gene therapies targeting the RPGR gene, such as those utilizing different vectors like AAV5 or incorporating novel technologies to control gene expression.

However, significant challenges remain in this clinical practice, primarily concerning the safety of therapies, longevity of effectiveness, and management of immune responses. One notable adversity is that nearly all participants in clinical trials experience complications such as conjunctival hemorrhage, ocular inflammation, or reduced visual clarity. These issues are primarily attributed to the surgical procedures involved in subretinal or intravitreal delivery rather than the gene therapy itself [73,74].

Moreover, selecting the most suitable AAV vector and determining an effective sample size remain crucial challenges. The variety of RPGR mutations, combined with differences in tropism and transduction efficiency across AAV serotypes, complicates the optimization process [48,50,51,72,73]. For instance, while AAV8 has demonstrated promise, its immune profile and specific tropism may not be universally advantageous. The emergence of novel capsid designs, such as AAV7m8 or AAV5, offers opportunities to refine these therapies (Table 2) [31,72,73].

Another critical hurdle is the prevalence of pre-existing anti-AAV antibodies in approximately 30–60% of the population. These antibodies can neutralize viral vectors and significantly limit the patient population eligible for treatment [77]. Addressing this obstacle may require innovative strategies, such as capsid engineering to evade neutralization or pre-treatment protocols to deplete circulating antibodies.

Finally, the high cost of gene therapies poses a significant barrier to widespread adoption. During the experimental phase, the estimated cost per patient is approximately USD 1 million, highlighting concerns about affordability and accessibility. However, it is essential to recognize that this issue is not unique to RPGR gene therapies; it reflects a broader challenge in the field of advanced therapeutics [78]. Continued advancements in vector production and distribution are likely to reduce costs over time, making these life-changing treatments more accessible.

In addition, the long-term durability of gene therapy for XLRP, as well as gene therapies in general, also remains a critical concern. As an emerging and rapidly evolving field, the long-term effectiveness of these treatments is still uncertain due to their cutting-edge nature and the rarity of the conditions they target. In most clinical trials conducted in recent years, post-treatment monitoring has typically spanned only 12 months, making it premature to draw definitive conclusions about the prolonged therapeutic effects of these therapies without robust, long-term evidence [70,71,72,73]. Nevertheless, the outcomes of these trials have been highly promising. These findings provide compelling evidence of the therapy’s efficacy, fostering optimism about its potential for durable and sustained benefits over time.

## 6. Conclusions and Future Perspectives

Recent advancements in gene therapy for XLRP have ushered in a new era of optimism for patients and clinicians alike. The use of AAV vectors to deliver functional copies of the RPGR gene has not only demonstrated promising results in preclinical models but has also translated into significant improvements in visual function and retinal structure in early-stage clinical trials. These breakthroughs highlight the potential of gene therapy to fundamentally alter the disease trajectory of XLRP.

Notably, clinical trials involving therapies such as AGTC-501 and AAV5-RPGR have shown encouraging outcomes, with patients experiencing improvements in retinal sensitivity and visual function [71,72,73,74]. These developments signify a critical step forward, bringing us closer to effective treatments that can preserve and restore vision in individuals affected by XLRP.

While challenges remain, such as ensuring long-term efficacy and safety, optimizing gene delivery to target cells, and addressing immune responses, the rapid progress in this field fuels hope that these obstacles will soon be overcome. Advances in vector design and delivery methods are enhancing specificity and minimizing off-target effects. Moreover, the genetic heterogeneity of XLRP, once a formidable challenge, is increasingly being addressed through personalized therapeutic approaches and improved understanding of genotype–phenotype correlations [10,79]. For instance, studies are beginning to unravel the complexities of mutations within the RPGR and RP2 genes [4,5,6,20,80] and efforts to establish robust genotype–phenotype relationships are gaining momentum [3,4,6,81].

Emerging technologies like CRISPR-Cas9 gene editing offer the promise of precisely correcting RPGR mutations, potentially providing a one-time, curative treatment [15]. Additionally, combining gene therapy with other modalities such as neuroprotectants or retinal prosthetics may yield synergistic effects, further enhancing therapeutic outcomes.

In conclusion, the horizon is bright for XLRP patients. Ongoing and future clinical trials are poised to validate these innovative treatments and establish standardized protocols. Collaborative efforts between researchers, clinicians, and regulators are accelerating the translation of these scientific advances into effective and accessible therapies for patients with XLRP [3]. With continued dedication and interdisciplinary collaboration, we are on the cusp of bringing new, life-changing treatments into the clinic, offering renewed hope to those affected by this challenging condition.

## Figures and Tables

**Table 1:** The table summarizes all registered clinical studies performed in the last five years (2019–2024) on X-linked retinitis pigmentosa (XLRP). The studies are divided into several phases (Phase I, II, III).

Therapeutics	Company/ University	Clinical Identifier (Phase)	Date and Status	Purpose
AAV8-RPGR	Biogen	NCT03116113 (I/II)	2017–2020, completed	posted
AAV2/5-RPGR	MeiraGTx UK II Ltd.	NCT03252847 (I/II)	2017–2021, completed	submitted
BIIB112 (AAV8-RPGR)	NightstaRx Ltd., a Biogen Company	NCT04926129 (Observational)	2017–2022, completed	no posted
AAV5 hRKp.RPGR	Janssen Research & Development, LLC	NCT06646289 (II)	2024–2030, Not yet recruiting	Safety and tolerability
FT-002	Frontera Therapeutics	NCT06492850 (I/II)	2024–2026 Recruiting	Safety, tolerability, and efficacy
rAAV2tYF-GRK1-RPGR	Beacon Therapeutics	NCT06333249 (II)	2021–2027 Active, not recruiting	Safety and efficacy
rAAV2tYF-GRK1-RPGR	Beacon Therapeutics	NCT06275620 (II)	2023–2029 Enrolling by invitation	Safety
AAV5-hRKp.RPGR	Janssen Pharmaceutical K.K.	NCT05926583 (III)	2023–2030 Recruiting	Safety, tolerability
FT-002	Frontera Therapeutics	NCT05874310 (I)	2023–2027 Recruiting	Safety, tolerability, and preliminary efficacy
rAAV2tYF-GRK1-RPGR	Beacon Therapeutics	NCT03316560 (I/II)	2018–2025 Active, not recruiting	Safety and efficacy
rAAV2tYF-GRK1-hRPGRco (AGTC-501)	Beacon Therapeutics	NCT04850118 (II/III)	2024–2029 Recruiting	Safety, efficacy, and tolerability
AAV5-hRKp.RPGR	Janssen Research & Development, LLC	NCT04794101 (III)	2020–2029 Active, not recruiting	Safety
AAV5-hRKp.RPGR	Janssen Research & Development, LLC	NCT04671433 (III)	2020–2024 Active, not recruiting	Safety
4D-125	4D Molecular Therapeutics	NCT04517149 (I/II)	2020–2029, Active, not recruiting	Evaluate natural disease progression, safety and tolerability
AAV5-hRKp.RPGR	Janssen Research & Development, LLC	NCT04312672 (Observational)	2017–2026 Active, not recruiting	Safety up to 60 months
AAV2-REP1, previously treated AAV8-RPGR	NightstaRx Ltd., a Biogen Company	NCT03584165 (III)	2018–2026 Active, not recruiting	Long-term safety and efficacy

**Table 2:** Pharmacologic strategies for the treatment of XLRP using AAV encoding RPE65 or RPGR.

Drug	Comments	Side Effects	Results	Ref.
LUXTURNA (voretigene neparvovecrzyl, AAV2-hRPE65v2)	AAV2 vector containing human RPE65 cDNA with a modified Kozak sequence engineered at the translational start site, under control of a hybrid chicken β-actin promoter with a cytomegalovirus enhancer.	Common: conjunctival hyperemia, cataract, increased intraocular pressure, retinal tear, dellen (thinning of the corneal stroma), macular hole, subretinal deposits, eye inflammation, eye irritation, eye pain, and maculopathy (wrinkling on the surface of the macula).	65% of patients were responded on therapy. Full-field stimulus testing (FST) and baseline at years 1 and 3: −18.41 dB at year 1 and −14.73 dB at year 3. Visual acuity remained stable without decrease, and visual function showed improvement in patients with chorioretinal atrophy over the 3-year period. MAIA at 12 months: 4.1 dB of gene therapy and 0 without therapy.	[69,70]
AGTC-501 (rAAV2tYF-GRK1-RPGR)	rAAV2tYF-GRK1-RPGR expresses the human RPGR^ORF15^ driven by the GRK1 promoter and packaged in AAV2 capsids with single tyrosine to phenylalanine (YF) mutations.	Not registered in human	63% of eyes treated with high dose were responded on therapy. Retinal Sensitivity at 12 months: >7 dB in at least five loci. MAIA 12 months: 1.96 dB of gene therapy and −0.39 dB of without therapy.	[71]
AAV5- RPGR (Botaretigene Sparoparvovec, AAV2/5.hRKp.RPGR)	AAV2/5.hRKp.RPGR is based on the AAV2/5 serotype but the *RPGR* sequence is mutated in the ORF15 region and carries a random deletion of 126 codons.	96.9% participants in the treatment groups reported an AE considered related to surgery. These AEs were transient and resolved without intervention: conjunctival hemorrhage, reduced VA, and the presence of anterior chamber cells	~50% of patients treated with AAV5-RPGR demonstrated significant improvements in visual function. Retinal sensitivity at 12 months: 0.95 dB of gene therapy and –0.59 dB of without therapy. MAIA: 12 months: 5.6 dB of gene therapy and–1.2 dB of without therapy. Functional vision assessment at 9 months: 16.4” in gene therapy with no errors and 61.7” with 2 errors of baseline. (”—seconds)	[72]
BIIB112 (Cotoretigene toliparvovec, AAV8-RPGR)	AAV8-RPGR vector containing the codon-optimized human RPGR^ORF15^ under the control of the GRK1 promoter	94% experienced 35 TEAEs associated with study procedure: 67% inflammation, Serious TEAEs 28%: visual acuity reduced, noninfective retinitis, retinal detachment, visual impairment.	~33% of patients treated with BIIB112demonstrated significant improvements in visual function. Retinal Sensitivity at 12 months: 2.8 dB of gene therapy and 0.1 dB of without therapy. MAIA 12 months: +5.1 dB of gene therapy and –0.9 dB of without therapy.	[74,75]

## Abbreviations

AAV	Adeno-associated virus
AEs	Adverse effects
ARL3	ADP-ribosylation factor-like GTPase 3
CME	Cystoid macular edema
dB	Decibels
GAP	GTPase-activating protein
GEF	Guanine nucleotide exchange factor
GRK1	G protein-coupled receptor kinase 1
IRBP	Interphotoreceptor retinoid-binding protein
iPSCs	Induced pluripotent stem cells
KO	Knockout
LCA	Leber congenital amaurosis
MAIA	Macular integrity assessment
nAbs	Neutralizing antibodies
ONL	Outer nuclear layer
PAM	Protospacer adjacent motif
PDE δ	Phosphodiesterase δ
RGCs	Retinal ganglion cells
rAAV	Recombinant AAV
ROS	Retinal organoids
RPE	Retinal pigment epithelium
RP	Retinitis pigmentosa
RPGR	Retinitis pigmentosa GTPase regulator
ROS	Reactive oxygen species
sgRNA	Single guide RNA
TEAEs	Treatment-emergent adverse events
TLRs	Toll-like receptors
UNC119	Uncoordinated 119 protein
VF	Visual field
VEGF	Vascular endothelial growth factor
XLRP	X-linked retinitis pigmentosa
